# A case report of abdominal metastatic dermatofibrosarcoma protuberans misdiagnosed as gastrointestinal stromal tumor

**DOI:** 10.1186/s13000-023-01430-9

**Published:** 2024-02-22

**Authors:** Minying Deng, Qingxiao Liu, Lei Ren, Wei Yuan, Chen Xu, Yingyong Hou

**Affiliations:** Department of Pathology, Zhongshan Hospital, Fudan University, Shanghai, 200032 China

**Keywords:** Dermatofibrosarcoma protuberans, Abdominal metastatic, Gastrointestinal stromal tumor

## Abstract

Dermatofibrosarcoma protuberans (DFSP) is a low-grade malignant soft-tissue tumor that originates from the skin. It has a slow onset in the early stages, non-specific clinical symptoms, low specificity, and can easily be overlooked, missed, or misdiagnosed by clinicians and pathologists. In addition, DFSP is prone to recurrence after local surgical treatment; however, distant metastasis, especially abdominal metastasis, is rare, which is also a challenge for the accurate diagnosis of DFSP when it progresses distantly. Now a case of abdominal metastasis of DFSP is reported. The patient has been treated with imatinib for ten years, and the lesion has shrunk, but because the patient has been receiving imatinib treatment, his abdominal lesion was once misdiagnosed as gastrointestinal stromal tumor. Therefore, we report on this case to enhance the understanding of the diagnosis and treatment of DFSP, and to provide reference for the pathological diagnosis and precise treatment of such patients.

## Case introduction

The patient, a 43-year-old female, claimed to have discovered a bottle-cap sized tumor next to her right knee when she was 10 years in primary school. The tumor did not change much and was surgically removed when she was 19 years old in 1999. She claimed that the postoperative pathological diagnosis was fibroma. In 2001, a bottle-cap sized tumor appeared again near the right knee joint. It was surgically removed when the patient was 23 years old in 2003. According to the patient, the pathological report was borderline fibroma. In 2005, the tumor near the right knee joint recurred again, but it was not treated. In 2008, when the patient was 28 years old and pregnant, she found that the tumor grew faster to the size of an egg, and the skin color on the surface of the tumor darkened, but she still did not pay attention to it. In June 2009, due to a knock, the tumor ruptured and was surgically removed. The postoperative pathological diagnosis was neurofibroma. In 2010, the patient developed a peanut-sized tumor on the outside of her left thigh. The postoperative pathological diagnosis was dermatofibrosarcoma protuberans. In 2012, when the patient was 32 years old, a tumor in the anterior basal segment of the right lower lobe was found due to hemoptysis. The postoperative pathology showed leiomyosarcoma. After two months of chemotherapy, a mass in the hilum was found again and removed. The postoperative pathology was still diagnosed as leiomyosarcoma. In March 2013, a 10 cm tumor in the abdomen was found due to abdominal distension and pain. It was surgically removed, and the postoperative pathological diagnosis was gastrointestinal stromal tumor (GIST). In October 2013, the tumor in the abdomen recurred, about 10 cm in size. She took 5–6 tablets of imatinib per day. According to her, the tumor shrunk to 5 cm after 3 months of medication. On February 9, 2023, a reexamination of CT showed that the size of the abdominal tumor was 6.1 × 4.6 × 4.2 cm. It was surgically removed. The immunohistochemical testing and pathological analysis of the outside hospital are as follows: (abdominal tumors and spleen) spindle cell soft tissue tumor, tumor cells only have moderate positive expression of a-SMA, Desmin and H-caldesmon are both negative, which does not support the diagnosis of leiomyosarcoma. Although CD34, CD117, DOG-1 are all negative, considering that the patient has been treated with imatinib for ten years, GIST is firstly considered.

**Fig. 1:**
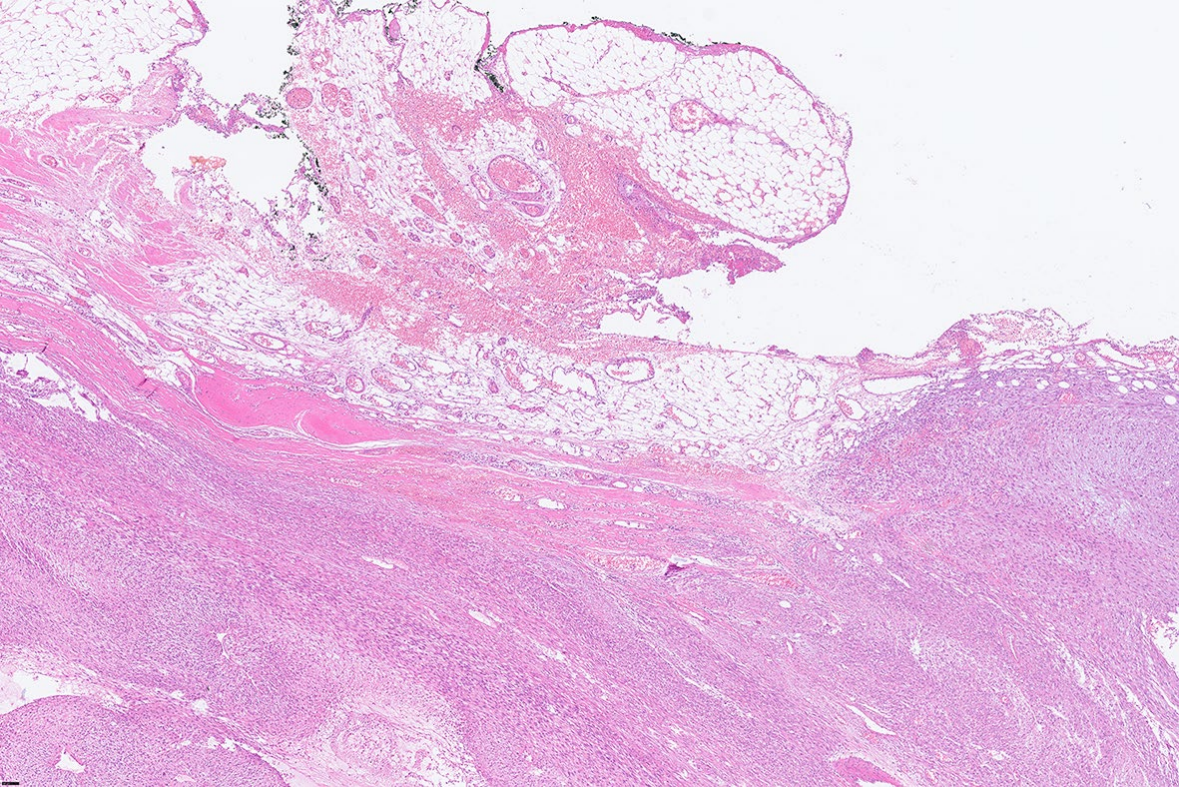
DFSP with unclear borders with the surrounding area, HE middle magnification

**Fig. 2:**
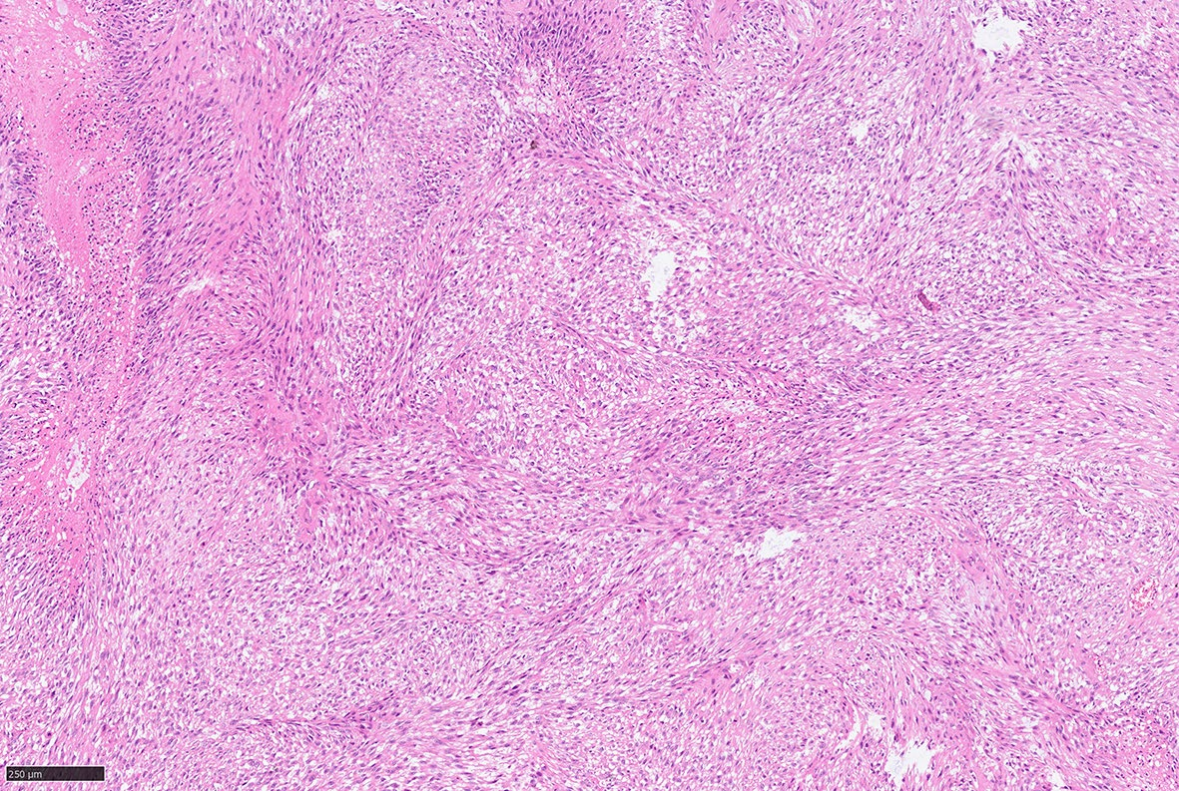
DFSP diffusely arranged in sheets, HE middle magnification

**Fig. 3:**
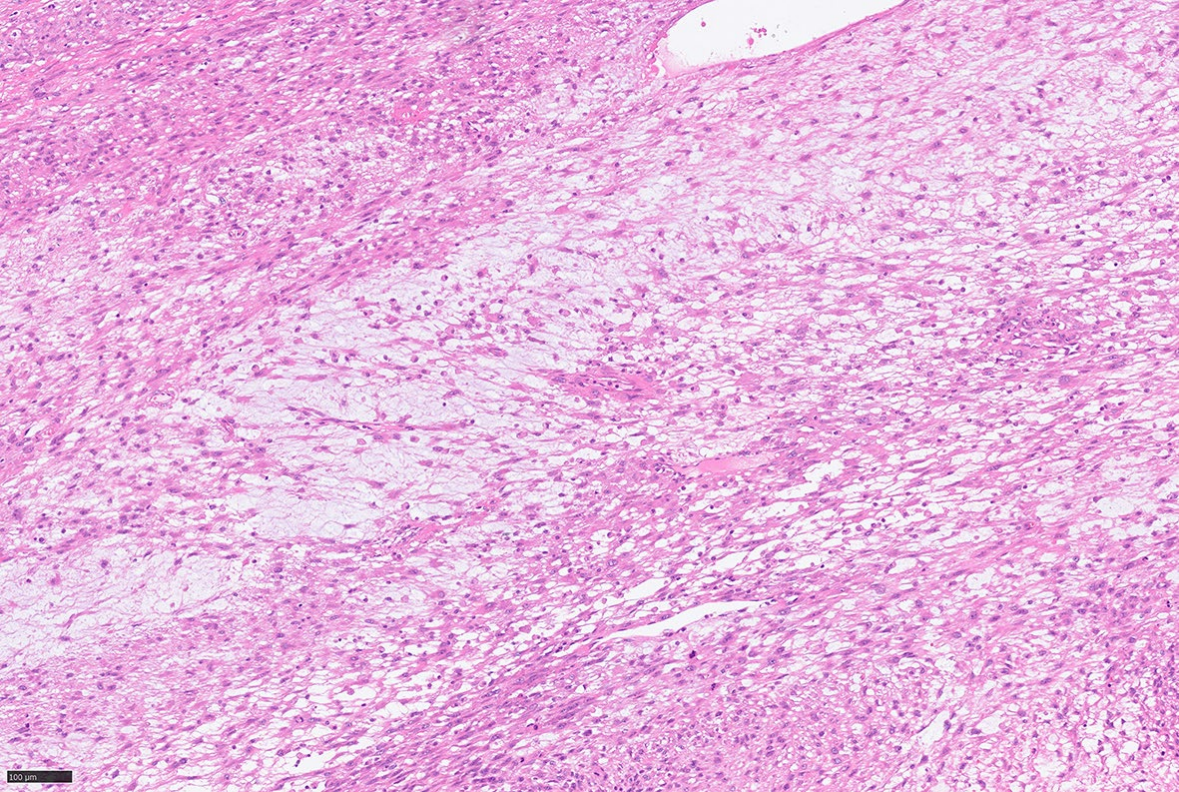
Mucinous changes can be seen in parts of DFSP, HE middle magnification

**Fig. 4:**
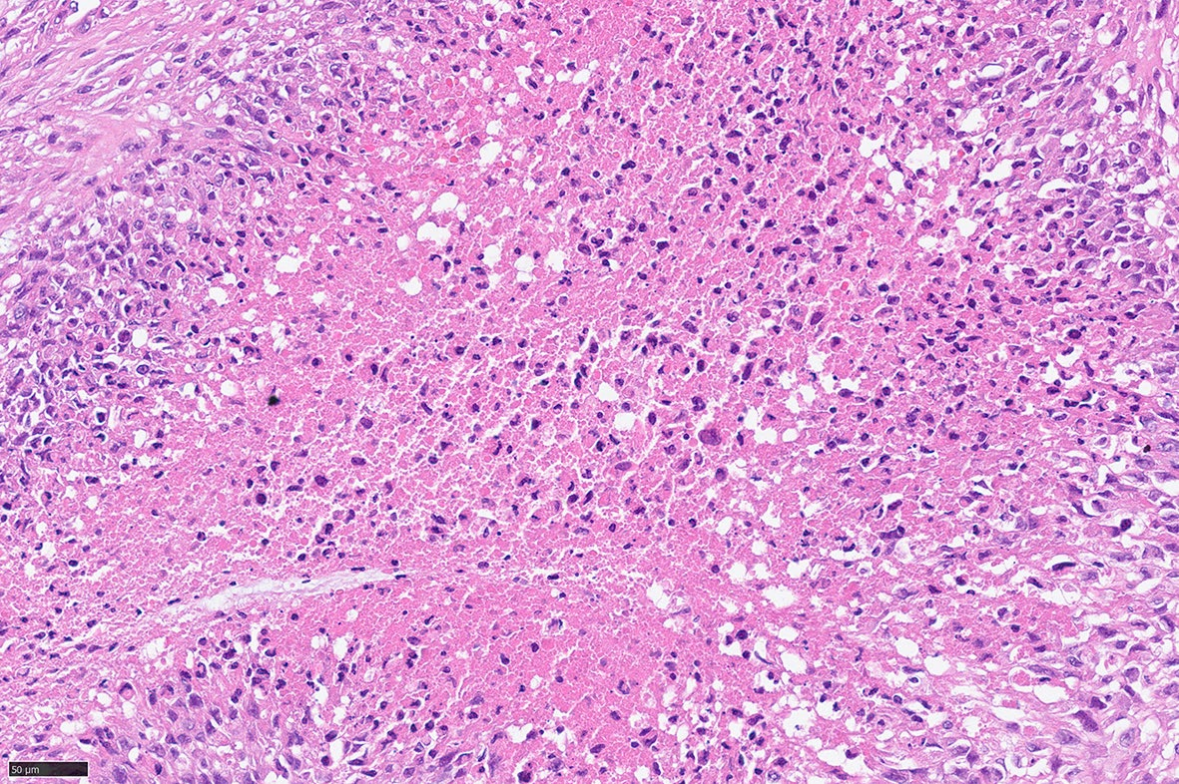
Necrosis can be seen in parts of DFSP, HE high magnification

**Fig. 5:**
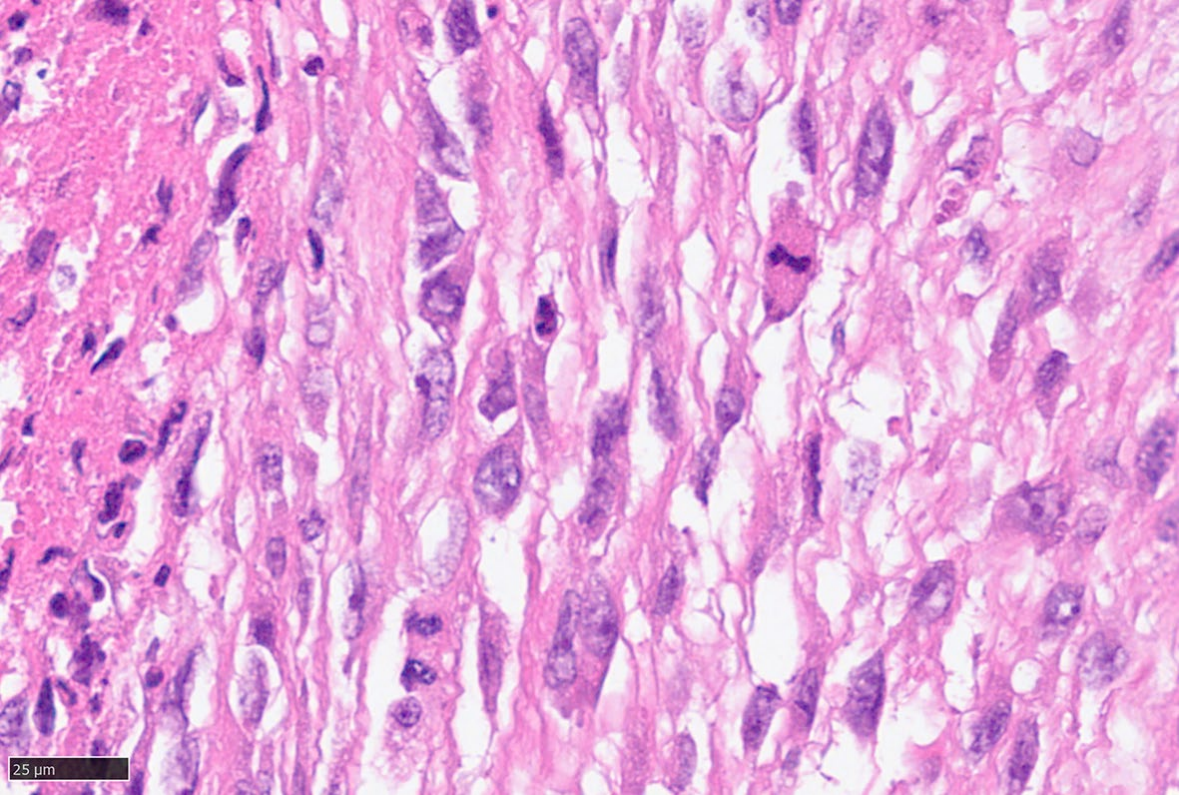
Easy to see nuclear division, HE high magnification

**Fig. 6:**
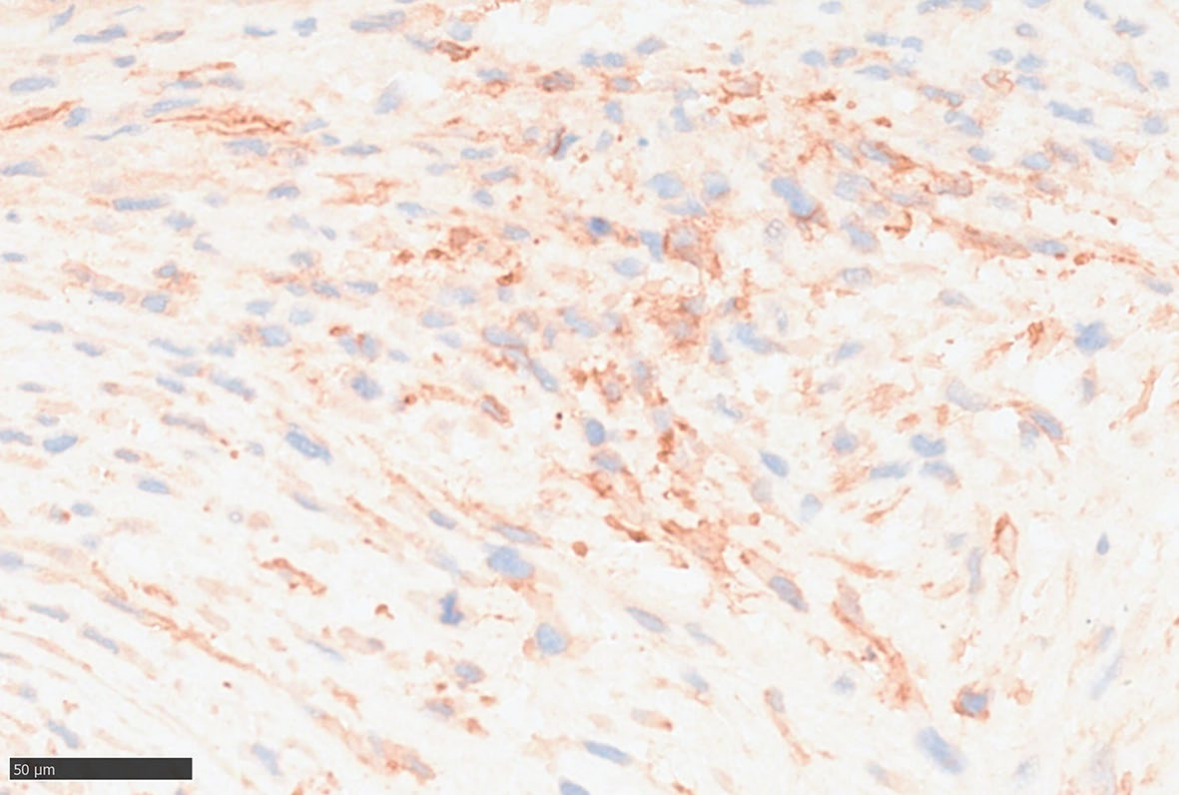
Tumor cells express a small amount of CD34, EnVision method, high magnification

**Fig. 7:**
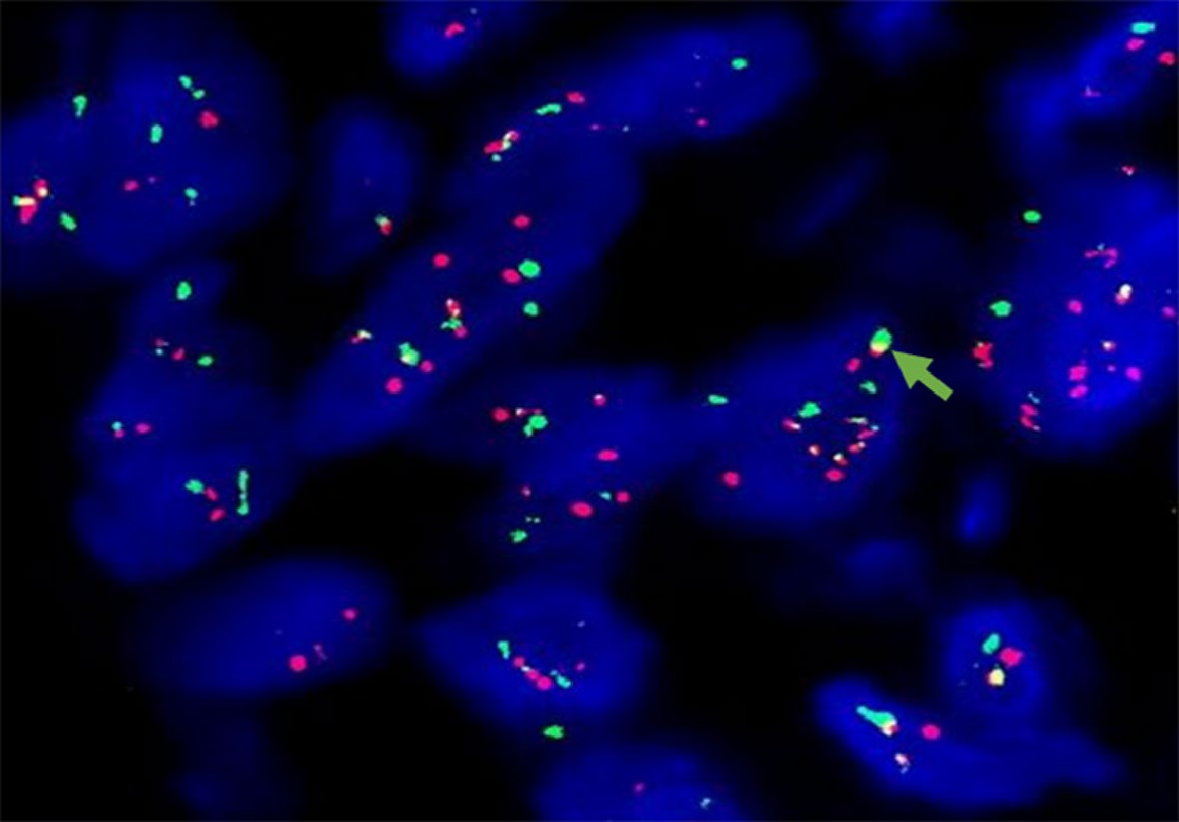
COL1A1-PDGFB fusion probe, yellow fusion signal can be seen, indicating the existence of COL1A1-PDGFB gene fusion, FISH method ×1000

**Fig. 8:**
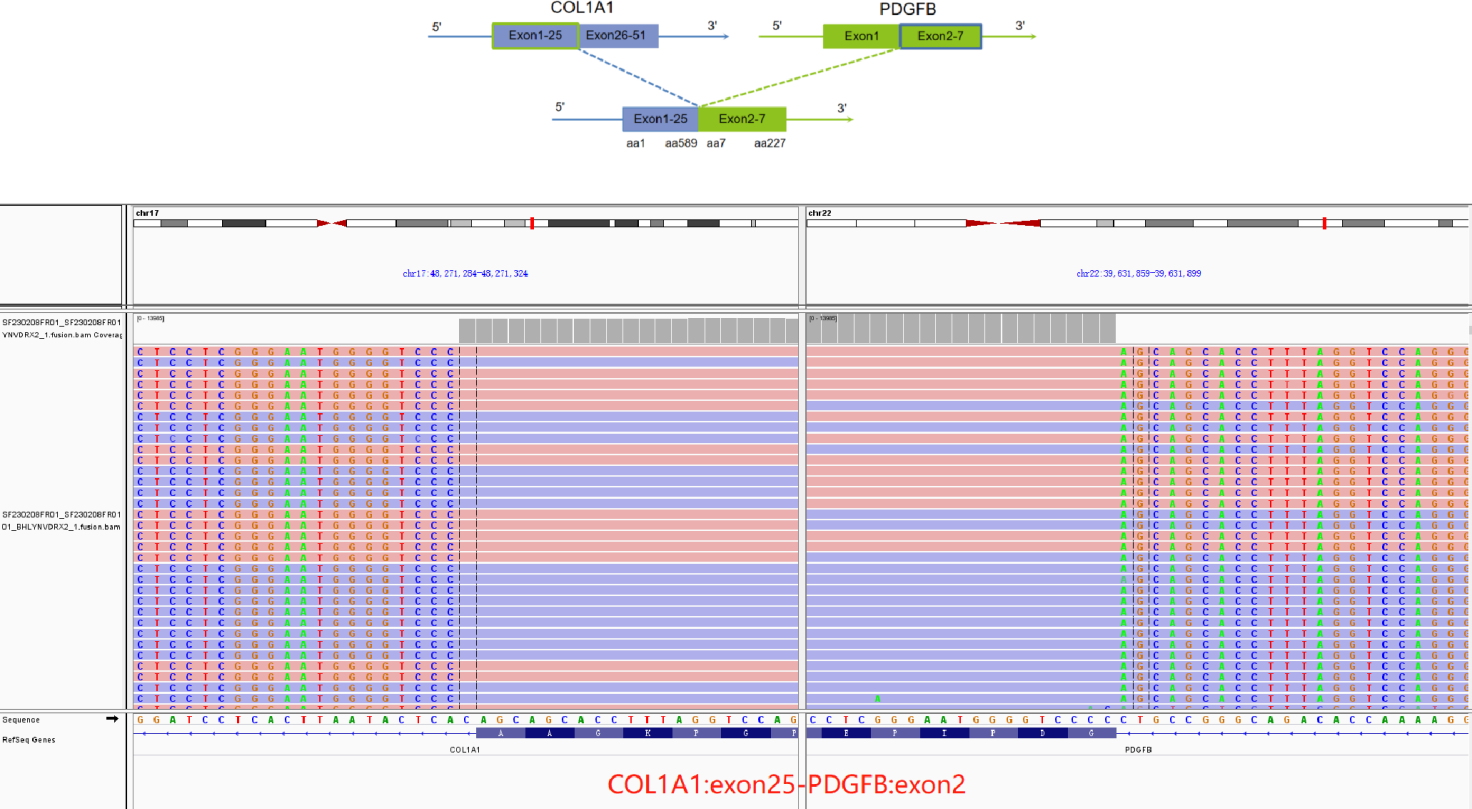
High-throughput sequencing shows COL1A1 (exon 25) -PDGFB (exon 2) fusion

For further accurate diagnosis and treatment, the patient brought the relevant materials of the abdominal tumor to our department of pathology for further consultation in April 2023. Under low magnification, the boundary between the tumor and the surrounding tissues is unclear, the tumor grows diffusely, tumor necrosis can be seen, and some stroma is mucinous. Under high magnification, the tumor cells are short spindle-shaped, with rich, eosinophilic cytoplasm, some of which contain vacuoles. The nuclei are round or oval, with fine chromatin, and some have small nucleoli. Nuclear fission is easy to see (Figs. 1, 2, 3, 4, 5).Immunohistochemical results show that the tumor cells diffusely express Nestin, a-SMA, SDHB, a small amount of CD34, and CD117, DOG-1, Calponin, h-Caldesmon, Desmin, S-100 and pan-cytokeratin (CKpan) are all negative. The Ki67 positive index is about 20% positive (Fig. 6). Fluorescence in situ hybridization technique detected COL1A1-PDGFB fusion gene, and high-throughput sequencing technique detected PDGFRA amplification, COL1A1-PDGFB fusion gene, FGFR1 gene exon 12 mutation, FGFR4 gene exon 10 mutation (Figs. 7, 8).

Our department’s pathological diagnosis: abdominal tumor with spindle cell malignant tumor, rich cells, mild to moderate dysplasia, nuclear fission of 49/50HPF, combined with medical history and gene detection results, is consistent with dermatofibrosarcoma protuberans abdominal metastasis.

Follow-up until September 26, 2023, the patient is still taking 6 tablets of imatinib per day, regular check-ups, no recurrence/metastasis, and the situation is good.

## Discussion

DFSP is a low-grade malignant skin soft tissue tumor with local invasiveness, occurring in the dermis and subcutaneous tissue, and some cases can evolve into more malignant fibrosarcomas. The tumor has a history of over a hundred years. In 1890, Taylor [1] first reported the disease, describing it as a rare, scar-like nodule-like tumor with a tendency to recur. In 1924, Daire [2] first fully described the clinical features of DFSP and named it “progressive recurrent skin fibroma”. In 1925, Hoffmann [3] officially named it Dermatofibrosarcoma Protuberans. The tumor is relatively rare, accounting for about 0.1% of all malignant tumors and only 1% of soft tissue tumors. According to statistics, the incidence of DFSP in the American population is 4.3/1,000,000, and the incidence in African-Americans is twice that of whites [4, 5]. The disease is more common in adults aged 30–50 years, slightly more common in males, and a few cases can occur in infants or children, accounting for about 3.7% [6]. The affected areas are often the trunk (40–50%), proximal limbs (30–40%), and head and neck (10-15%), and can also occur in less common areas such as the perineum [5, 6]. The occurrence of the tumor may be related to a history of trauma, surgery, immunization, and other factors. The onset is slow, and the disease course can last for decades. It has been reported that the tumor can grow rapidly during pregnancy [7]. DFSP rarely metastasizes, less than 5%, and metastasis often occurs after multiple recurrences, most commonly to the lungs, and rarely to lymph nodes.

The patient in this case was a middle-aged female who had nodules at the extremities since childhood. As the time span was quite long, it was difficult to review the slides of the surgery to remove the nodules at the extremities. She had a history of rapid enlargement of a tumor near the right knee during pregnancy. However, the first diagnosis of DFSP was made at the proximal extremities, i.e., the lateral thigh. In summary, due to the complexity of the patient’s history and limited detection technology or means at the time, it is speculated that the patient might have had the disease since childhood, and it has recurred multiple times and metastasized to the lungs and abdominal cavity, evolving into a relatively late-stage case, which is rare and peculiar.

Generally, DFSP presents as patchy thickening or nodular changes on the skin surface. The primary tumor is usually solitary, while recurrent tumors are often multifocal. The cut surface of the tumor is grayish-white and rubbery, the texture is mostly hard, and some are slightly soft due to mucoid changes, necrosis is relatively rare.

Under the microscope, the tumor is mostly located in the dermis, and short spindle cells can be seen arranged in patches and diffusely distributed, showing characteristic storiform or cartwheel patterns, often infiltrating into subcutaneous adipose tissue. However, not all tumors present classic DFSP morphological changes. According to the 5th edition of the WHO classification of soft tissue and bone tumors in 2020, DFSP can also present as the following pathological subtypes: (1) Pigmented DFSP: Also known as Bednar tumor, it is characterized by varying numbers of scattered dendritic pigmented cells. It can be differentiated from benign fibrous histiocytoma or diffuse/pigmented neurofibromas by positive CD34 expression in tumor cells; (2) Fibrosarcomatous DFSP: In some cases, tumor cells show severe atypia, visible mitotic figures, and appear as elongated bundles or fishbone-like arrangements, similar to fibrosarcoma. A small number of cases can transform into pleomorphic undifferentiated sarcoma, which should be distinguished; (3) Myxoid DFSP: Some areas show myxoid changes, which need to be differentiated from other myxoid soft tissue tumors. For example, myxoid liposarcoma can detect DDIT3 gene translocation by fluorescence in situ hybridization technique, while PDGFB is negative; (4) DFSP with muscular/myofibroblastic differentiation: Eosinophilic muscular nodules or bundles can provide certain hints for diagnosing this subtype; (5) Atrophic or plaque-type DFSP: Generally presents as skin atrophy, loose skin, or plaques, and the tumor is located in the superficial dermis, making it easy to confuse with dermatofibroma or fibrosis. Other rare pathological subtypes have also been reported, such as hybrid tumors, sclerotic DFSP, lattice-like and Verocay body-containing DFSP, DFSP with extensive meningothelial-like whorls, and granular cell DFSP [8–12]. In this case, the tumor was misdiagnosed as fibroma, neurofibroma, leiomyosarcoma, and even gastrointestinal stromal tumor during the initial stages of local recurrence, indicating that the tumor is prone to be misdiagnosed as other soft tissue tumors.

Immunohistochemical results showed that tumor cells diffusely express CD34, p75, and partially express EMA (cytoplasm), while S-100, Desmin, AE1/AE3, actins, FXIIIa all tested negative. DFSP can also express GRIA2, with a positivity rate of 75% [13]. Recent reports show that WT1 has a positive expression rate of 95% in DFSP, located in the cytoplasm. Because CD34 has high sensitivity (94%) but not strong specificity (83%), researchers believe that the discovery of WT1 can complement CD34 in diagnosing DFSP [14]. In addition, 5-hydroxymethylcytosine (5hmC) is valuable in distinguishing DFSP from dermatofibroma, and its low expression may increase the invasiveness of DFSP [15]. IGFBP7 and Stromelysin-3 can serve as negative immunohistochemical markers for DFSP, and when combined with CD34 and FXIIIa, they can make the diagnosis of DFSP more credible [16]. However, when the malignancy of DFSP increases, such as in fibrosarcomatous DFSP and myxoid DFSP, the expression of CD34 is significantly reduced, which should be given attention. In this case, only a small amount of CD34 is positive, which can easily be confused if relying solely on immunohistochemical markers. This case reminds pathologists to make comprehensive judgments combining the patient’s history and other factors to provide the most accurate diagnosis.

The molecular pathogenesis of DFSP is relatively clear. Studies have shown that over 90% of cases are characterized by the characteristic t (17; 22) (q22; q13) chromosomal translocation and the formation of additional ring chromosomes r (17; 22) due to t (17; 22). Both mechanisms can form the COL1A1-PDGFB fusion gene. Under normal conditions, the PDGFB gene is suppressed, but the COL1A1 in the fusion gene can provide a promoter and signal peptide for PDGFB. The fusion protein produced by COL1A1-PDGFB is then processed into mature PDGFB, which interacts with the PDGFB receptor (platelet-derived growth factor receptor-β, PDGFR-β) on the surface of DFSP, leading to dysfunction of PDGF, activation of downstream signaling pathways RAS-MAPK and PI3K-AKT-mTOR, and ultimately promoting the proliferation of DFSP cells [17, 18]. Some researchers have conducted large-scale (53 cases) whole-genome sequencing of DFSP for the first time, suggesting that the SLC2A5-BTBD7 fusion gene may be a new potential diagnostic and therapeutic target for DFSP, and detected AKT1 and SPHK1 oncogene amplification and CDKN1A/B gene expression deficiency [19]. Of course, other rare molecular genetics have also been reported, such as EMILIN2-PDGFD fusion, COL1A2-PDGFB fusion, COL6A3-PDGFD fusion, etc. [20–22].

Currently, the US Food and Drug Administration has approved imatinib mesylate for the treatment of unresectable, metastatic or advanced dermatofibrosarcoma protuberans. Imatinib mesylate is a small molecule tyrosine kinase inhibitor, a PDGFR-β, ABL, c-KIT blocker, which can interfere with the phosphorylation of receptor tyrosine kinase (RTKs) and the activation of downstream pathways by competitively binding with adenosine triphosphate (ATP), thereby blocking the PDGFB signaling pathway. In Rutkowski’s study [23], there was no significant difference in the efficacy of 400 mg/d and 800 mg/d doses for treating DFSP, but the 400 mg/d dose had fewer side effects. Therefore, starting with a dose of 400 mg/d is recommended. This result was also adopted by a European consensus guideline, which proposed a treatment approach of gradually increasing the dose from a low dose (400-600 mg/d) to a higher dose (600-800 mg/d) [24]. Relevant data showed that in neoadjuvant treatment of DFSP with imatinib mesylate, a dose of 600 mg/d resulted in an average tumor reduction of 31.5% after a mean treatment duration of 3.1 months, with partial response reaching 50% [25]. Kérob et al. [26] demonstrated that preoperative treatment with imatinib mesylate (600 mg/d) for 2 months resulted in a median volume reduction of 20% in approximately 36% of cases. The study by Ugurel et al. [25] also suggested that a dose of 600 mg/d of imatinib mesylate in neoadjuvant treatment for DFSP was effective and well-tolerated with manageable adverse reactions. A study on Chinese DFSP patients showed that the initial dose of 400 mg was increased to 800 mg due to the development of imatinib mesylate resistance. After an average treatment of 15 months, the 1-year and 3-year overall survival rates were 95.5% and 77.3%, respectively, indicating that imatinib mesylate is safe and well tolerated in the treatment of DFSP [27]. However, only more than 50% of DFSP respond to imatinib mesylate, and resistance or secondary resistance can still not be avoided, which is speculated to be related to weak PDGFRB phosphorylation or the COL1A1-PDGFB fusion gene [25]. Therefore, some researchers believe that sunitinib may have good efficacy in treating DFSP patients who fail imatinib mesylate treatment, possibly related to the fact that sunitinib’s binding ability with PDGFRB is ten times that of imatinib mesylate [28, 29]. In this case, regardless of whether the pathological diagnosis is GIST or DFSP, the dose of imatinib mesylate is 5–6 tablets per day, which is consistent with the literature reports.

DFSP has a high misdiagnosis rate and is prone to local recurrence after surgery, up to 60%. Therefore, regular follow-up examinations are extremely important for preventing DFSP recurrence and metastasis. However, current research provides different recommendations for follow-up. In 2015, the European consensus suggested follow-up every 6 months for the first 5 years, followed by annual follow-up for the next 5 years, totaling 10 years of follow-up. Recurrent DFSP and DFSP with fibrosarcomatous transformation should undergo imaging examinations during follow-up, especially for DFSP with fibrosarcomatous transformation, as lung metastasis commonly occurs and routine lung monitoring is recommended [24, 30]. Studies have shown that the median time from surgical excision to local recurrence is 32–38 months. Therefore, Bowne et al. [31] recommended follow-up examinations every 6–12 months. Hao et al. [32] proposed comprehensive history collection and clinical examinations of the primary site and draining lymph nodes at each visit. Further imaging examinations should be considered based on the tumor size, location, position, growth rate, surgical procedures, and histopathology (presence of high-risk factors and margin status). Biopsy should be performed for suspected local or distant recurrence or lymph node metastasis. A study from the United States suggested that low-risk patients (e.g., tumor ≤ 5 cm, no fibrosarcomatous changes, R0 surgical excision) can perform self-examinations under physician guidance and follow-up education, while high-risk patients should be followed up for at least 10 years [33]. The NCCN guidelines recommend follow-up every 6–12 months for the primary site and regular imaging examinations for patients with high-risk features [34]. In summary, although researchers have not fully agreed on follow-up methods and timing, the focus is mainly on the tumor’s primary site and its surrounding areas.

In this case, the patient had a history of long-term treatment with imatinib mesylate, and after taking it for 3 months, the tumor volume reduced by 50%, with good results. The initial use of imatinib mesylate for treatment was due to a misdiagnosis of gastrointestinal stromal tumor, because both are related to PDGF gene mutations, making it difficult to make the most accurate diagnosis for over ten years. Therefore, this case’s pathological diagnosis process is worth reflecting on by pathologists: First, for relatively inert tumors, which may experience several recurrences and last for several decades, multiple pathologists may diagnose the disease, with each pathologist possibly only encountering a certain stage, often overlooking the entire medical history and the patient’s past history. Second, while DFSP is a classical disease, its incidence is low, and it presents challenges for accurate diagnosis due to its complex pathological subtypes and rare occurrence of metastasis. There are not a few patients with multiple primary diseases, and the initial fibroma was naturally excluded from the relationship with this case, leading to a later misdiagnosis as GIST, also naturally ignoring the entire medical history, thus increasing the interference factors for an accurate diagnosis of this disease. Third, we may not be familiar with the tumor spectrum of imatinib treatment, and pathologists have a deep understanding that imatinib is a molecular target drug for GIST, but the most fundamental principle of imatinib is that it is a drug for the tyrosine kinase activity of BCR-ABL1, KIT, PDGFRA, etc. It was first used for chronic myeloid leukemia, was later found to be used for GIST, and can also be used for DFSP. However, we cannot infer in reverse that all soft tissue tumors treated with imatinib targeting molecular therapy are GIST, which is an area where we modern pathologists need to learn and reflect in order to make an accurate diagnosis. As pathologists, our goal is to provide accurate diagnoses and treatment recommendations for patients. Therefore, we need to continuously learn and update our knowledge system in order to better understand the molecular mechanisms and targeted treatment options for different tumor types. This means that we need to closely collaborate with clinical physicians to stay informed about the latest treatment strategies and drug advancements. At the same time, we also need to reflect on and review our diagnostic criteria and standards to ensure that we can accurately identify and differentiate various tumor types. This includes emphasizing the research and learning of various rare tumors in tumor pathology education to improve our understanding and diagnostic proficiency of these diseases. The patient in this case is still using imatinib mesylate for treatment, but high-throughput sequencing technology has detected PDGRFA amplification, and perhaps subsequent use of sunitinib will have a certain effect. In addition, high-throughput sequencing technology has detected FGFR1 and FGFR4 gene mutations in DFSP for the first time, which have not been reported in the literature, and their specific mechanisms of action need further study. PDGRFA mutations are often detected by fluorescence in situ hybridization, but now high-throughput sequencing technology can also detect specific mutation sites such as exons and introns, which are of great help in discovering hidden COL1A1‑PDGFB fusion or rare fusion.

In summary, DFSP is rare, with a low degree of malignancy, and abdominal metastasis is even rarer. This case was misdiagnosed as a gastrointestinal stromal tumor and was treated with imatinib mesylate, which suggests that pathologists should make comprehensive judgments based on medical history, immunohistochemistry, and molecular genetic results. It also suggests the importance of detecting the COL1A1-PDGFB fusion gene in DFSP using methods such as FISH or high-throughput sequencing technology, and hopes to provide some experience for clinicians in treating DFSP through this case.

## Data Availability

Not applicable.
